# Comprehensive bronchoalveolar lavage characterization in COVID-19 associated acute respiratory distress syndrome patients: a prospective cohort study

**DOI:** 10.1186/s12931-023-02464-9

**Published:** 2023-06-09

**Authors:** Fiorella Calabrese, Francesca Lunardi, Elisa Baldasso, Federica Pezzuto, Asuman Kilitci, Gheorghe-Emilian Olteanu, Claudia Del Vecchio, Francesco Fortarezza, Annalisa Boscolo, Marco Schiavon, Luca Vedovelli, Annamaria Cattelan, Dario Gregori, Federico Rea, Paolo Navalesi

**Affiliations:** 1 Department of Cardiac, Thoracic, Vascular Sciences, and Public Health, University of Padova Medical School, Padova, Italy; 2 Pathological Anatomy Unit, Padova University Hospital, Padova, Italy; 3 Department of Medicine, University of Padova Medical School, Padova, Italy; 4 Microbiology and Virology Unit, Padova University Hospital, Padova, Italy; 5 Department of Medical Pathology, Faculty of Medicine, Düzce University, Düzce, Turkey; 6 Department of Infectious Diseases, Discipline of Pulmonology, Center for Research and Innovation in Personalized Medicine of Respiratory Diseases, “Victor Babes” University of Medicine and Pharmacy, Timisoara, Romania; 7 Center of Expertise for Rare Lung Diseases, Clinical Hospital of Infectious Diseases and Pneumophisiology “Dr. Victor Babes”, Timisoara, Romania; 8 Institute of Anaesthesia and Intensive Care, Padova University Hospital, Padova, Italy; 9 Thoracic Surgery Unit, Padova University Hospital, Padova, Italy; 10 Infectious Diseases Unit, Department of Medicine, Padova University Hospital, Padova, Italy

**Keywords:** ARDS, Bronchoalveolar lavage, COVID-19, Cytokine profile, Microbiology

## Abstract

COVID-19-related acute respiratory distress syndrome (CARDS) is associated with high mortality rates. We still have limited knowledge of the complex alterations developing in the lung microenvironment. The goal of the present study was to comprehensively analyze the cellular components, inflammatory signature, and respiratory pathogens in bronchoalveolar lavage (BAL) of CARDS patients (16) in comparison to those of other invasively mechanically ventilated patients (24). In CARDS patients, BAL analysis revealed: SARS-CoV-2 infection frequently associated with other respiratory pathogens, significantly higher neutrophil granulocyte percentage, remarkably low interferon-gamma expression, and high levels of interleukins (IL)-1β and IL-9. The most important predictive variables for worse outcomes were age, IL-18 expression, and BAL neutrophilia. To the best of our knowledge, this is the first study that was able to identify, through a comprehensive analysis of BAL, several aspects relevant to the complex pathophysiology of CARDS.

**Supplementary Information:**

The online version contains supplementary material available at 10.1186/s12931-023-02464-9.

## Introduction

The vast spectrum of clinical manifestations of SARS-CoV-2 infection ranges from asymptomatic or paucisymptomatic forms to severe pneumonia with acute respiratory distress syndrome (ARDS) requiring often admission to an intensive care unit (ICU) [1] with in-ICU mortality ranging in Europe from 28 to 42% [2]. COVID-19-associated ARDS (CARDS) has often been associated with rapid virus replication, inflammatory cell infiltration, and elevated cytokines resulting in multiorgan failure, mainly pulmonary [3, 4]. We still have limited knowledge of the complex alterations developing in the lung microenvironment of patients with CARDS. Most studies have used blood, plasma, and/or serum [5–7], while only few were performed in BAL samples reporting heterogeneous results [8–12]. A comprehensive comparative study with appropriate controls allowing for inflammatory and infection profiling of BAL has not yet been done.

Accordingly, the goal of the present study was to compare cellular components, inflammatory signatures, and the main pathogens in BALs collected from CARDS patients and two different control groups of invasively mechanically ventilated (IMV) patients (healthy and frail patients) drawn from the same ICU. An additional exploratory goal was to identify the most important factors that discriminate worse outcomes.

## Materials and methods

**Fig. 1:**
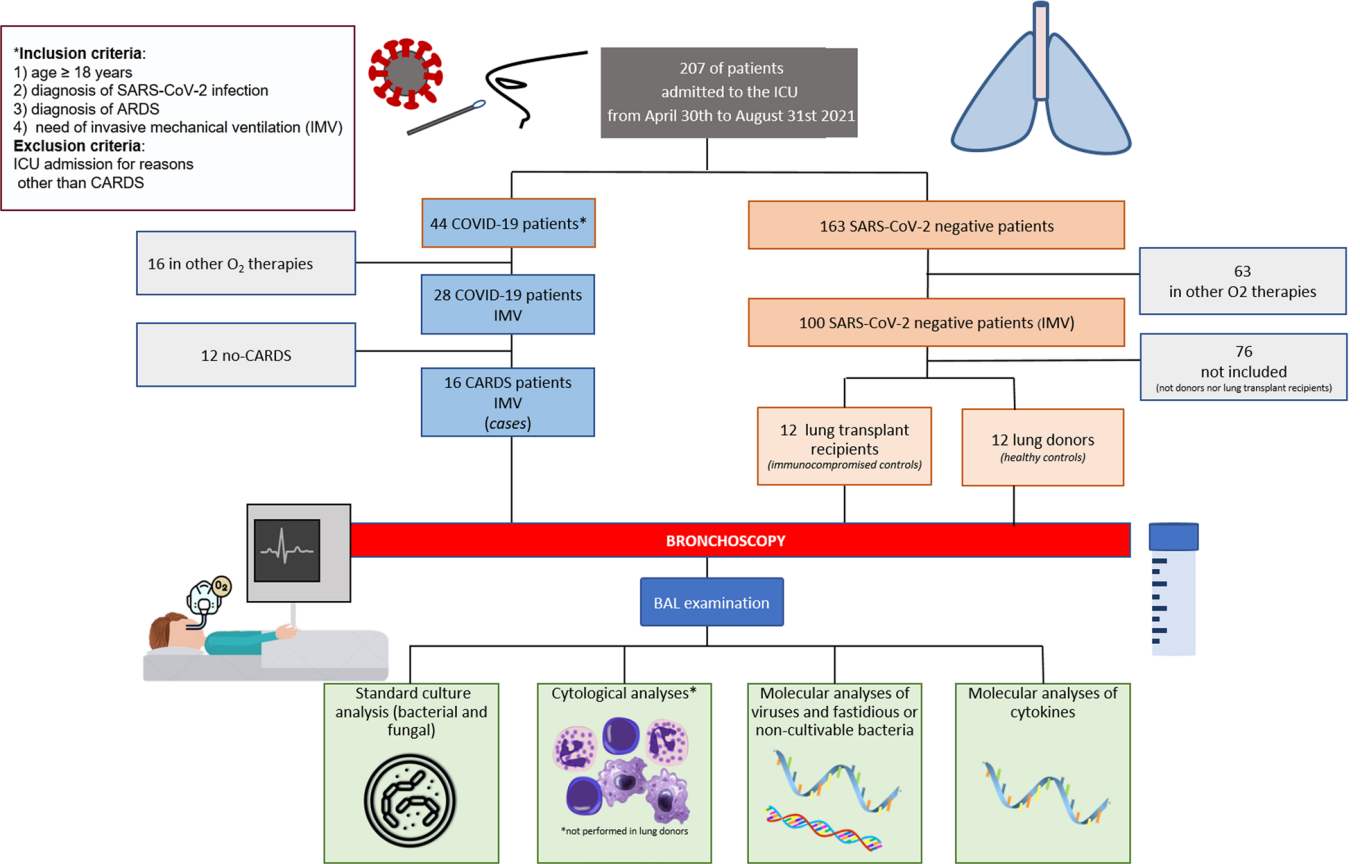
CONSORT diagram describing the study population and design

We performed a prospective single-center cohort study enrolling 16 CARDS cases admitted to the ICU of Padova University Hospital between April 30th and August 31st, 2021, following specific inclusion and exclusion criteria (Fig. 1).

Two different control groups were used: frail controls: *“immunocompromised” (IC) controls* including lung transplant (LT) recipients (n = 12); and *“healthy” controls* including lung donors (n = 12). SARS-CoV-2 RT-PCR was carried out and cases with sufficient viral load (< 27 Ct) were sequenced using a Genetic Analyzer (Applied Biosytems, Foster City, CA, USA). All patients in the control groups were negative for SARS-CoV-2 and recruited in the same time interval as that of CARDS. BAL from *healthy controls* was negative for any kind of infection (so called “sterile” BAL) and collected within three days after ICU admission. BALs from *IC controls and* from all *CARDS cases* were collected within two weeks after ICU admission. The study was approved by the Institutional Ethics Committee of Padova (number: 5245/AO/21; April 15th, 2021) and was conducted in accordance with the principles of the Declaration of Helsinki. Informed consent was obtained according to national regulations. All investigations were performed on de-identified data. For each *CARDS case* and each *IC control*, demographic characteristics, clinical and laboratory data, medical treatments, ICU/hospital length of stay, ICU/hospital mortality, and radiological data were collected in electronic medical records (Table 1, original datasets at 10.25430/researchdata.cab.unipd.it.00000694). Details on BAL processing for microbiological, cytological and molecular analyses of inflammatory mediators, as well as the statistical analyses performed, can be found in the Additional file 1.

**Table 1:** Clinical characteristic of study population at enrollment

	CARDS cases (n = 16)	IC controls (n = 12)	Healthy controls (n = 12)
*Gender*			
Male, n	12 (75%)	8 (67%)	6 (50%)
Female, n	4 (25%)	4 (33%)	6 (50%)
Age, years	60 [51–73]	57 [46–61]	53 [39–58]
BMI, Kg/m^2^	31 [27–40]	25 [22–27]	24 [20–26]
SOFA score	5 [5–6]	n.a.	n.a.
*Comorbidities* ^*a*^			
Diabetes, n	5 (31%)	3 (25%)	0 (0%)
Hypertension, n	5 (31%)	2 (17%)	0 (0%)
COPD, n	2 (12%)	0 (0%)	0 (0%)
Chronic kidney disease, n	2 (12%)	0 (0%)	0 (0%)
Chronic liver disease, n	1 (6%)	0 (0%)	0 (0%)
Active neoplasms, n	1 (6%)	0 (0%)	0 (0%)
No significant comorbidities, n	6 (38%)	7 (58%)	12 (100%)
*Laboratory data*			
White blood cells (x10^9^/L)	10 [8–13]	6 [6–9]	12 [11–13]
Neutrophils (x10^9^/L)	8 [6–10]	4 [3–6]	6 [5–7]
Lymphocytes (x10^9^/L)	0.8 [0.7– 1.1]	2 [1–2]	1.3 [1.1–1.4]
C-reactive protein (mg/ml)	86 [42–140]	17 [15–27]	35 [16–65]
*In-hospital terapies*			
Prolonged corticosteroids, n	16 (100%)	12 (100%)^b^	0 (0%)
Antiviral, n	3 (19%)	12 (100%)	0 (0%)
Antibacterial, n	16 (100%)	12 (100%)	0 (0%)
Antimycotic, n	9 (56%)	12 (100%)	0 (0%)
Anticoagulants, n	16 (100%)	12 (100%)	0 (0%)
Anakinra, n	2 (13%)	0 (0%)	0 (0%)
*Outcomes*			
ICU LOS, days	21 [15–29]	5 [4–6]	4 [4–5]
H LOS, days	32 [29–60]	36 [32–41]	4 [4–5]
ICU mortality, n	5 (31%)	0 (0%)	–
H mortality, n	5 (31%)	0 (0%)	–

Data are expressed as number and (percentage) or as median and [IQR]

*IC* immunocompromised (lung transplant recipients), *BMI* body mass index, *SOFA* Sequential Organ Function Assessment, *COPD* chronic obstructive pulmonary disease, *ICU* intensive care unit, *H* hospital, *LOS* length of stay, *IQR* interquartile range, *n* number, *n.a.* not available

^a^Some patients had 2 or more comorbidities

^b^Immune suppressive therapy, usually combined to basiliximab and tacrolimus

## Results

**Fig. 2:**
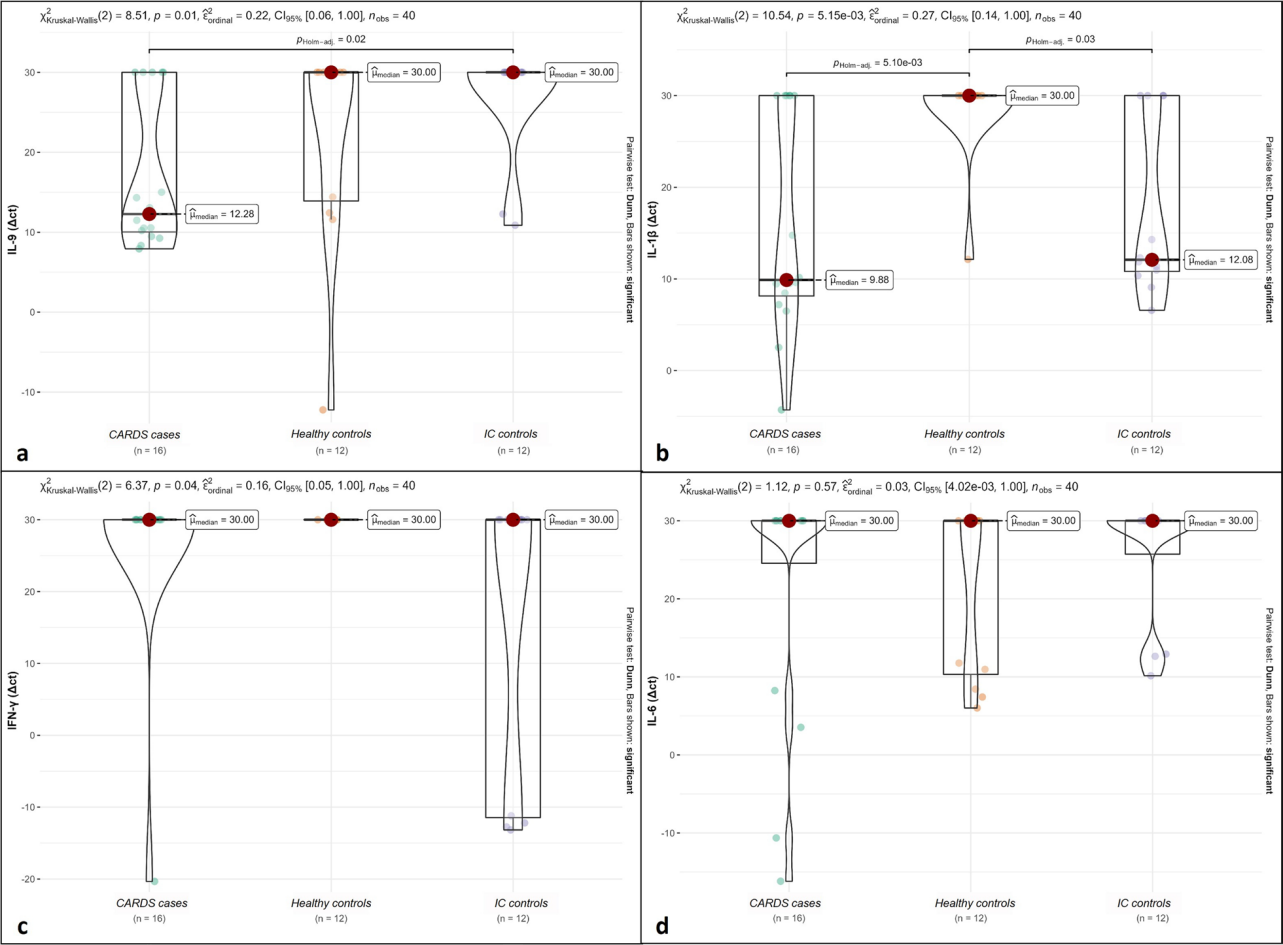
Comparison of the median ΔCt values of IL-9 (**a**), IL-1β (**b**), IFN-γ (**c**), and IL-6 (**d**) in *CARDS patients*, *IC and healthy controls*. IFN-γ was less expressed in *CARDS patients* than *IC controls* (equally to healthy controls) (p = 0.04) while IL-9 and IL-1β were significantly more expressed in *CARDS cases* than in *IC controls* and* healthy controls* (p = 0.01 and p = 0.005). No statistical significance was achieved for IL-6

While gender, age, and BMI are not different in the three groups, *CARDS cases* show a slightly higher BMI and hypertension prevalence (Table 1). Real-time PCR for SARS-CoV-2 was positive in all BALs of *CARDS cases* with a mean of 27.65 CT (range from 16 to 35). SARS-CoV-2 sequence analysis resulted in variant Alpha. All BALs from *controls* were negative. At least one bacterial isolate was detected in 5 (31%) *CARDS cases* and in 5 (42%) *IC controls*. Fungi were found in 5 (31%) *CARDS patients* and in none of the *IC control group*. As expected, no cultivable bacteria or fungi were detected in BALs from *healthy control*s (Table 2). Molecular analysis of viruses and no cultivable bacteria revealed viral infection in 5 BALs (31%) from *CARDS cases* and in 5 (42%) from the *IC controls* (Table 2). None of the *healthy controls* were positive for the microorganisms investigated by molecular analyses (Table 2). Cytological analysis of BALs from *CARDS cases* were frequently rich in mucus and showed high cellularity, mainly neutrophils (15/16,94%). Neutrophilic granulocyte percentage was significantly increased in BAL samples from *CARDS cases* (median %, IQR: 55, 40–90 vs. 0, 0-2.25; p < 0.001) while lymphocytic and macrophagic values were significantly lower than in the *IC control group* (median %, IQR: 0, 0–0 vs. 5, 4.5–10 for lymphocytes, 40, 10–50 vs. 90, 90-93.5 for macrophages). Reactive pneumocytes, fibrin and blood were present in almost all *CARDS cases* (87.5%, 100% and 93.8%, respectively). Molecular expression analysis of inflammatory mediators showed significant differences in the expression of IL-9, IL-1β, IFN-γ, IFN-α7 and IFN-α8 (Table 3; Fig. 2). In particular, IFN-γ was less expressed in *CARDS patients* than *IC controls* (equal to *healthy controls*) (p = 0.04) while IL-9 and IL-1β were significantly more expressed in *CARDS cases* than in *IC controls* and *healthy controls* (p = 0.01 and p = 0.005). Some explanatory cases are presented in the Additional file 2 and 3. Statistical analysis showed that BAL neutrophil granulocyte percentage of *CARDS cases* was higher independently from the presence of concomitant infection, hospital LOS and ICU LOS. Similarly, no difference in cytokine expression was detected when comparing *CARDS patients* with or without coinfections. All cellular components, cytokine profile, and infectious agents of *CARDS cases* seem not to be associated with clinical data such as age, sex, BMI, ICU, and hospital LOS (using the Spearman non-parametric correlation test). The Boruta algorithm using all cytokine quantification, blood tests, bacterial, viral, and fungal infections, age, gender, and BMI as predictors showed that the most important variables determining the outcome were: age for mortality, no variable for ICU stay, IL-18 for hospital LOS, and BAL neutrophil granulocytes percent in case of ECMO.

**Table 2:** Microbiological findings of the study population

	CARDS cases (n = 16)	IC controls (n = 12)	Healthy controls (n = 12)
Bacterial isolates (N)			
None	11 (69%)	7 (58%)	12 (100%)
* Klebsiella pneumoniae*	1 (6%)	1 (8%)	0 (0%)
* Klebsiella pneumoniae* ESBL	1 (6%)	0 (0%)	0 (0%)
* Pseudomonas aeruginosa* MDR	0 (0%)	2 (17%)^b^	0 (0%)
Enterobacter cloacae	1 (6%)	1 (8%)	0 (0%)
* Staphylococcus aureus*	2 (13%)	2 (17%)^b^	0 (0%)
Fungal isolates (N)			
None	11 (69%)	12 (100%)	12 (100%)
* Candida albicans*	4^a^ (25%)	0 (0%)	0 (0%)
* Aspergillus fumigatus*	2^a^ (13%)	0 (0%)	0 (0%)
Viruses (N)			
None	11 (69%)	7 (58%)	12 (100%)
EBV	1 (6%)	2 (17%)^c^	0 (0%)
HSV1	4 (25%)	4 (33%)^c^	0 (0%)
CMV	0 (0%)	2 (17%)^c^	0 (0%)

*ESBL* extended beta-lactamase, *MDR* multi drug resistant, *EBV* Ebstein Barr virus, *HSV* Herpes Simplex virus, *sp* species, *CMV* cytomegalovirus, *n* number

^a^In 1 patient: Aspergillus + Candida

^b^In 1 patient: Klebsiella + Staphylococcus

^c^In 3 patients, two viruses were detected: HSV1 + CMV, EBV + CMV, EBV + HSV1

**Table 3:** Inflammatory mediator profiles

Inflammatory mediators	CARDS cases (n = 16)	IC controls (n = 12)	Healthy controls (n = 12)	p-value^a^
IFN-α1	7 (6, 8)	9 (7, 11)	7 (6, 7)	0.065
IFN-α16	12 (10, 30)	30 (26, 30)	11 (11, 30)	0.2
IFN-α17	8.7 (7.9, 10.6)	9.4 (8.4, 10.6)	8.2 (7.4, 9.0)	0.13
IFN-α2	9 (8, 10)	9 (9,11)	8 (8, 9)	0.2
IFN-α6	7.7 (7.0, 9.0)	8.2 (7.4, 9.2)	7.0 (6.6, 7.5)	0.2
**IFN-**α**7**	7.21 (6.86, 9.03)	9.35 (8.14,10.00)	7.19 (6.89, 7.82)	**0.024**
**IFN-α8**	9 (8, 10)	9 (8, 10)	8 (7, 8)	**0.038**
IFN-β1	8 (8, 10)	8 (8, 10)	8 (7, 9)	0.3
**IFN**-**γ**	30 (30, 30)	30 (-11, 30)	30 (30, 30)	**0.041**
IL10	30 (30, 30)	30 (30, 30)	30 (26, 30)	0.2
IL-12α	30 (30,30)	30 (30, 30)	30 (30, 30)	0.7
IL-12β	30 (21, 30)	30 (30, 30)	30 (30, 30)	0.4
IL-13	30 (30, 30)	30 (30, 30)	30 (30, 30)	0.094
IL-15	30 (30, 30)	30 (30, 30)	30 (30, 30)	> 0.9
IL-16	30 (30, 30)	22 (12, 30)	30 (30, 30)	0.3
IL-17α	30 (30, 30)	30 (30, 30)	30 (30, 30)	0.3
IL-18	30 (30, 30)	30 (26, 30)	30 (30, 30)	0.2
IL-1 A	30 (25, 30)	30 (12, 30)	30 (30, 30)	0.068
**IL-1β**	10 (8, 30)	12 (11, 30)	30 (30, 30)	**0.005**
IL-2	30 (30, 30)	30 (30, 30)	30 (30, 30)	0.5
IL-3	30 (30, 30)	30 (30, 30)	30 (30, 30)	0.5
IL-4	30 (30, 30)	30 (30, 30)	30 (30, 30)	0.2
IL-5	30 (30, 30)	30 (30, 30)	30 (30, 30)	0.7
IL-6	30 (25, 30)	30 (26, 30)	30 (10, 30)	0.6
IL-8	21 (8, 30)	12 (10, 30)	30 (13, 30)	0.3
**IL-9**	12 (10, 30)	30 (30, 30)	30 (14, 30)	**0.014**
LTA	30 (30, 30)	30 (30, 30)	30 (30, 30)	0.3
TNF	30 (16, 30)	30 (11, 30)	30 (30, 30)	0.2

Data are presented as median (IQR) ΔCt

*CARDS* COVID-19-related acute respiratory distress syndrome, *IC* immunocompromised, *IFN* interferon, *IL* interleukin, *LTA* lymphotoxin-alpha, *TNF* tumor necrosis factor

^a^Kruskal-Wallis rank sum test. Inflammatory mediators with significant difference among the groups are marked in bold

## Discussion

To the best of our knowledge, this is the first study identifying relevant aspects of *CARDS patients* through a comprehensive analysis of different BAL aspects (cellular components, inflammatory signature, and infections). Specifically concerning microbial investigation, interestingly we detected the SARS-CoV-2 viral genome in all BALs from *CARDS cases* including those with a longer history of disease. Several studies have found prolonged viral shedding in BAL from critically ill patients compared to upper-respiratory tract specimens [13]. This may be related to the lack of neutralizing antibodies, which favor a greater number of “free” virions unbound by immune complexes thus contributing to prolonged infectivity [13]. Although there is still conflicting data about viral load and outcome, the most recent evidence on large case series indicates higher mortality in patients with higher viral load [13] Data from a very recent study investigating the microbiome and host immune profile indicate that the abundance of SARS-CoV-2 in the lower airways associated with a low host immunological response is a predictive sign of mortality [13]. Many bacteria and rare viruses were detected in our *CARDS cases*, some of them like those of the *IC control*s, such as *Klebsiella pneumoniae, Staphylococcus aureus*, and viral genomes (i.e., *EBV* and *HSV1)*. Fungal infections were detected only in *CARDS cases*, which were responsible for COVID-associated pulmonary aspergillosis (CAPA). The incidence of CAPA in critically ill COVID-19 patients is estimated to be between 26.3 and 33% [14, 15]. An interesting recent study by Viciani et al. on the lung microbiome supports the evidence that lung fungal dysbiosis is more severe in CARDS [16]. Analysis of the cellular components and overall cytokine expression in our study revealed intriguing findings. BALs showed high cellularity with a neutrophilic pattern in many cases (in 55% of cases). It is noteworthy that the neutrophilic pattern was not influenced by bacterial and fungal infections. In line with our observations indicating increased BAL neutrophils as a worse predictive factor for ECMO, an increased number of hyperactivated neutrophils was recently found in BAL of critical COVID-19 patients that required IMV and/or ECMO [12]. Excessive neutrophil extracellular trap generation and higher frequencies of immature neutrophils with an immunosuppressive phenotype have been proposed as novel therapeutic targets in critical COVID-19 patients [17]. Comparative analysis of inflammatory cytokine levels showed a significant IFN-γ downregulation in *CARDS cases*, similarly to healthy controls. While this was an expected finding in the healthy group, given that the patients are infection-free, this should not have been the case in the *CARDS cases* where either high SARS-CoV-2 viral load or other superinfections were found. Considering the immune role played by IFN-γ, we expected IFN-γ to be at least present if not highly expressed. Few studies have evaluated IFN-γ expression in COVID-19 patients, and they presented conflicting results mainly in relation to the source of investigation (BAL vs. blood vs. swab) and the time of disease (early vs. late) [18–20]. Preservation of the IFN-γ response in the *IC controls* further supports the evidence of impairment of antiviral defenses associated with SARS-CoV-2 infection more than an impairment related to iatrogenic immunosuppression (steroid therapy). Investigations on the IFN family, particularly IFN-γ in patients with COVID-19 and particularly those with CARDS need an urgent and in-depth analysis due to its possible use as a theragnostic biomarker. Finally, our results show significantly overexpressed IL-1β and IL-9 in *CARDS cases*. A previous study that investigated several cytokines in BAL supporting our data and showing that the expression of different cytokines, including IL-1β, was significantly higher in severe and critically ill COVID-19 patients (6 patients) [7]. IL-9 was undoubtedly the most characterizing inflammatory cytokine in our study population, because it was over-expressed only in the *CARDS cases.* Feng et al. [21] evaluated the mechanistic role played by IL-9 in deep venous thrombosis and provided data that supports a pro-thrombosis role played by IL-9. Considering that one of the major complications of CARDS are thromboembolic injuries in the pulmonary vascular bed [22–24], this finding may provide a basis for IL-9 suppressive intervention in COVID-19 disease, especially in critically ill patients. In addition to high BAL neutrophil percentage as a predictive marker for ECMO, age was found as another important predictive variable for mortality, as consistently reported in the literature [2, 25–27]. The present research is limited by its relatively small sample size: this reflects the rapid decline in the incidence of SARS-CoV-2 infections during the study period compared to the major pandemic waves. However, one of the strengths of this study was the inclusion of two control groups (an “immunocompromised” group and a “healthy” group) recruited during the same interval time and in the same ICU, and the use of robust statistical techniques, the results of which are useful to better understand the pathophysiology of complex host/microenvironment interaction and to mitigate some influencing factors (e.g., immunosuppressive therapy, IMV). At the same time, we are aware that a robust analytical method can only mitigate the small number of cases, thus further studies are needed to assess the generalization of our findings. In summary, this prospective comprehensive comparative BAL investigation of CARDS showed relevant features: the persistence of SARS-CoV-2 associated with superinfections, the marked BAL neutrophilia, and a specific cytokine set. We strongly believe that our findings may contribute to a better understanding of the complex pathophysiology of CARDS. These findings need additional research studies to explore mechanistic pathways.

## Supplementary Information

**Additional file 1.** Additional details concerning Materials and Methods.

**Additional file 2.** Explanatory case 1, showing a high number of neutrophils (a, hematoxylin and eosin staining, 40x original magnification) in BAL of a *CARD patient* without superinfection. IFN-γ was not detected while IL1β and IL-9 were found by molecular analyses (b, c and d, respectively).

**Additional file 3.** Explanatory case 2, showing a high number of neutrophils (a, hematoxylin and eosin staining, 40x original magnification) in BAL of a *CARDS patient* with a concurrent Aspergillus infection and numerous hyphae well seen at high magnification with special stain (b, PAS staining, 40x original magnification). IFN-γ was not detected by molecular analyses (c).

## Data Availability

The datasets supporting the conclusions of this article are available in the repository 10.25430/researchdata.cab.unipd.it.00000694.

## Abbreviations

ARDS: Acute respiratory distress syndrome
BAL: Bronchoalveolar lavage
BMI: Body mass index
CAPA: COVID-associated pulmonary aspergillosis
CARDS: COVID-19-related acute respiratory distress syndrome
CMV: Cytomegalovirus
EBV: Epstein-Barr virus
ECMO: Extracorporeal membrane oxygenation
HSV: Herpes virus simplex
IC: Immunocompromised
ICU: Intensive care unit
IFN: Interferon
IL: Interleukin
IMV: Invasive mechanically ventilated
IQR: Interquartile range
LOS: Length of stay
LT: Lung transplant
RT-PCR: Real Time polymerase chain reaction
WHO: World Health Organization
