# Supplementary material for: Comprehensive bronchoalveolar lavage characterization in COVID-19 associated acute respiratory distress syndrome patients: a prospective cohort study
Source: Respir Res. 2023 Jun 9;24:152. doi: 10.1186/s12931-023-02464-9 (PMC10250841; doi:10.1186/s12931-023-02464-9)
Supplement: Supplementary file 1 — Additional file 1. Additional details concerning Materials and Methods. [file 12931_2023_2464_MOESM1_ESM.doc]

**Comprehensive bronchoalveolar lavage characterization in COVID-19 associated acute respiratory distress syndrome patients: a prospective cohort study**

# Additional file 1

Six patients (40%) were tracheostomized and five (31%) needed veno-venous extracorporeal membrane oxygenation. Prolonged corticosteroid treatment with different therapeutic regimens according to specific guidelines was used in *CARDS cases* and *IC controls*.^e1,e2^

Donors occasionally received steroid treatments, usually methylprednisolone (15 mg/kg iv).^e3,e4^

***Bronchoscopy and bronchoalveolar collection***

After intravenous administration of sedation agents and routine inspection of the tracheobronchial tree, BAL was performed with a fiberoptic bronchoscope in a wedge position within the selected broncho-pulmonary segment. The total instilled volume of normal saline was no less than 100 ml and did not exceed 300 ml.^e5^ For an optimal sampling of distal airspaces, the total volume (pooled aliquots) retrieved was kept greater than or equal to 30% of the total instilled.^e5^ Finally, the total retrieved volume was divided into two aliquots (usually 10-15 ml)^e6^, one of which was used for microbiological assays, and the other was stored for cytological analyses and molecular cytokine investigation. BAL samples from *healthy controls* were processed only for microbiological and cytokine evaluation. BAL samples obtained from both CARDS and control patients were not different in terms of volume or viscosity.

***Microbiological analysis of BAL fluid***

BALs were processed for standard culture analyses (bacterial, mycobacterial, and fungal) and molecular determination of viruses and fastidious or non-cultivable bacteria (in total 23 microorganisms investigated). For molecular analyses, the MagNA Pure Compact Nucleic Isolation Kit I (Roche Molecular Systems) was used to obtain both DNA and RNA following the manufacturer’s instructions.

SARS-CoV-2 RT-PCR was also carried out using primers and probes designed by Corman et al. ^e7^ Results with an adequate amount of viral load (< 27 Ct) were sequenced using a Genetic Analyzer (Applied Biosytems, Foster City, CA, USA).

The following viral or bacterial microorganisms were investigated using in-house RT-PCR 7900HT RT-PCR System (Applied Biosystems) validated for clinical use [Chlamydophila pneumonia, Herpes virus simplex (HSV) types 1 and 2, Epstein-Barr virus (EBV), Adenovirus, Mycoplasma pneumoniae, Bordatella pertussis, Cytomegalovirus (CMV), Coronavirus HKU1, Coronavirus NL63, Coronavirus UPE, Coronavirus 229, Coronavirus OC43, Parainfluenza virus types 1, 2, and 3, Influenza viruses A and B, Rhinovirus, and respiratory syncytial virus].

***Molecular analyses of inflammatory mediators***

The cDNA was synthesized from 500 ng of total RNA, using 50 μM Random Hexamers (Invitrogen, ThermoFischer Scientific, Watham, MA, USA), 10 mM dNTPs mix (Invitrogen, ThermoFischer Scientific, Waltham, MA, USA), RNase Inhibitor 20 U/µL (Applied Biosystems, ThermoFischer Scientific, Waltham, MA, USA), DTT (Invitrogen, Thermo Fisher Scientific, Walthman, MA, USA), 50 U/µL of Superscript IV Reverse Transcriptase (Invitrogen, Thermo Fisher Scientific, Waltham, MA, USA) in a final volume of 20 uL following the manufacturer’s protocol. The cDNA used for cytokine analyses was measured using NanoDrop OneC and then diluted to 100 ng/μl for each sample. 32 μl of each work solution were added to 328 μl of water and to 360 μl of TaqMan® Fast Advanced Master Mix (Applied Biosystems, Foster City, CA, USA); each of these new solutions was then used to load the array plate (20 μl/well). The human Cytokine Network 96-well Plate contains 28 assays to cytokine network associated genes and 4 assays to candidate endogenous control genes. All assays were plated in triplicate.

Cytokine gene expression was examined using TaqMan® Array Human Cytokine Network (Applied Biosystems) with predesigned human gene-specific primers and with probes based on published cytokine sequences and following the manufacturer’s instructions.

The average Ct was determined and then the ΔCt value was calculated by normalizing target genes with the 18S housekeeping gene. *IC* and *healthy controls* were used to calculate the relative transcript levels (fold-changes) as x = 2 ^-ΔΔCt^ in which ΔΔCt = ΔCt *(CARDS cases*) −ΔCt (*controls*).

Specimen processing, slide preparation, and cell count were performed according to the standard certified laboratory procedures and following the laboratory biosafety guidelines for handling and processing specimens associated with COVID-19 published by the CDC. ^e8^

***Cytological analysis of BAL fluids***

Cytological analysis was performed in all BALs for *CARDS* cases and *IC controls*. The BAL fluid recovery rate from *healthy controls* was not enough to allow an adequate cytological evaluation. At least three slides were prepared from each BAL sample stained with Giemsa, Papanicolaou, Gram, and Gomori-methenamine-silver, the last used to test for fungal elements. The cytopathological slides were read by at least three board-certified pathologists (F.C., F.P., A.K., E.G.O., F.F.). All cytological analyses included the evaluation of the following parameters: (i) BAL adequacy and representativeness, when the percentage of ciliated columnar cells was less than 5% ^e9^ (ii) quantification of inflammatory cellular component, (iii) identification of microorganisms with appropriate histochemical stains, and (iv) description of other components (fibrin, pneumocytes, blood).

***Statistical analysis***

Continuous data are reported as medians (with first and third quartile); categorical data are reported as a percentage and absolute frequencies. Wilcoxon rank-sum tests were performed for continuous variables and the Fisher’s exact test or Pearson chi-square test for categorical variables. All statistics were computed using ΔCt values, which are inversely related to the expression value of the target gene. The ΔCt values were compared to all available clinical and morphological data. The Kruskal-Wallis rank-sum test was used to compare cytokine expression data as a continuous variable (ΔCt). In the case of non-expression, a ΔCt value of 30 was used as a convention. Several clinical factors were considered as possible confounders for CARDS data interpretation and thus were investigated: age, co-infections, laboratory data (as lymphocyte count and D-dimers), hospital and ICU length of stay, BMI, and comorbidities.

Feature selection was implemented using a machine-learning algorithm based on random forest (Boruta). The Boruta algorithm aims to identify all the relevant predictors that affect the outcome of interest (in our study, mortality, need for VV-ECMO, or hospital and ICU length of stay).

It implements a random forest on an augmented set of covariates. Additional covariates, called shadow variables, are copies of the original ones obtained by permuting the observations and thus removing possible associations with the outcome. For each explanatory variable, an important measure was computed, i.e., the Z-score, which is the average improvement in the predictive performance of the random forest with the considered explanatory variable divided by its standard deviation. The resulting important predictors are those that show a Z-score higher than the one observed for the variable with the maximum Z-score among the shadow variables. The procedure is repeated until an important measure is assigned to each predictor or until the maximum number of random forests is reached. Since a concern was the small number of patients (and thus the probable instability of the selection algorithm), we restrained the selection procedure by repeating the analysis with five different seeds (with different lengths) and keeping only the variables that emerged in all the five iterations at different seeds. Significance for importance was set to p < 0.01 for robustness and p < 0.05 for the other tests. No multiple comparison correction was applied, since we designed the experiment as a hypothesis generator.^e10, e11^ The {Boruta} R package was used for the analysis. R 4.2.1 was used for the analysis and plotting (using {ggstatsplot} and {gtsummary} packages). The full analysis code and datasets are available at (http://doi.org/10.25430/researchdata.cab.unipd.it.00000694).

# 3. Additional figure legends

*Additional figure 1*

Explanatory case 1, showing a high number of neutrophils (a, hematoxylin and eosin staining, 40x original magnification) in BAL of a CARD patient without superinfection. IFN-γ was not detected while IL1β and IL-9 were found by molecular analyses (b, c and d, respectively)

*Additional figure 2*

Explanatory case 2, showing a high number of neutrophils (a, hematoxylin and eosin staining, 40x original magnification) in BAL of a *CARDS patient* with a concurrent Aspergillus infection and numerous hyphae well seen at high magnification with special stain (b, PAS staining, 40x original magnification). IFN-γ was not detected by molecular analyses (c).

# 4. References

e1. RECOVERY Collaborative Group, Horby P, Lim WS, et al. Dexamethasone in Hospitalized Patients with Covid-19. *N Engl J Med*. 2021;384:693-704. doi:10.1056/NEJMoa2021436

e2. https://www.covid19treatmentguidelines.nih.gov/.

e3. Kotloff RM, Blosser S, Fulda GJ, et al. Management of the Potential Organ Donor in the ICU: Society of Critical Care Medicine/American College of Chest Physicians/Association of Organ Procurement Organizations Consensus Statement. *Crit Care Med*. 2015;43:1291-1325. doi:10.1097/CCM.0000000000000958

e4. Copeland H, Hayanga JWA, Neyrinck A, et al. Donor heart and lung procurement: A consensus statement [published correction appears in J Heart Lung Transplant. 2020 Jul;39(7):734]. *J Heart Lung Transplant*. 2020;39:501-517. doi:10.1016/j.healun.2020.03.020

e5. Meyer KC, Raghu G, Baughman RP, et al. An official American Thoracic Society clinical practice guideline: the clinical utility of bronchoalveolar lavage cellular analysis in interstitial lung disease. *Am J Respir Crit Care Med*. 2012;185:1004-1014. doi:10.1164/rccm.201202-0320ST

e6. Sampsonas F, Kontoyiannis DP, Dickey BF, Evans SE. Performance of a standardized bronchoalveolar lavage protocol in a comprehensive cancer center: a prospective 2-year study. *Cancer*. 2011; 117:3424-3433. doi:10.1002/cncr.25905

e7 Corman VM, Landt O, Kaiser M, Molenkamp R, Meijer A, Chu DK, et al. Detection of 2019 novel coronavirus (2019-nCoV) by real-time RT-PCR. Euro Surveill. 2020;25:2000045.

e8 https://www.cdc.gov/coronavirus/2019-ncov/lab/guidelines-clinical-specimens.html#:~:text=Insert%20the%20entire%20collection%20tip,seconds%20to%20collect%20the%20specimen.

e9 Meyer KC, Raghu G, Baughman RP, Brown KK, Costabel U, du Bois RM, et al. An official American Thoracic Society clinical practice guideline: the clinical utility of bronchoalveolar lavage cellular analysis in interstitial lung disease. Am J Respir Crit Care Med. 2012;185:1004-14.

e10 Saville, D.J. Multiple Comparison Procedures: The Practical Solution. *The American Statistician.* 1990;44,174-180.

e11 Rothman KJ. No adjustments are needed for multiple comparisons. *Epidemiology*. 1990;1:43-6.
