# Supplementary figures and images for: Comprehensive bronchoalveolar lavage characterization in COVID-19 associated acute respiratory distress syndrome patients: a prospective cohort study
Source: Respir Res. 2023 Jun 9;24:152. doi: 10.1186/s12931-023-02464-9 (PMC10250841; doi:10.1186/s12931-023-02464-9)

# Additional Figure 1


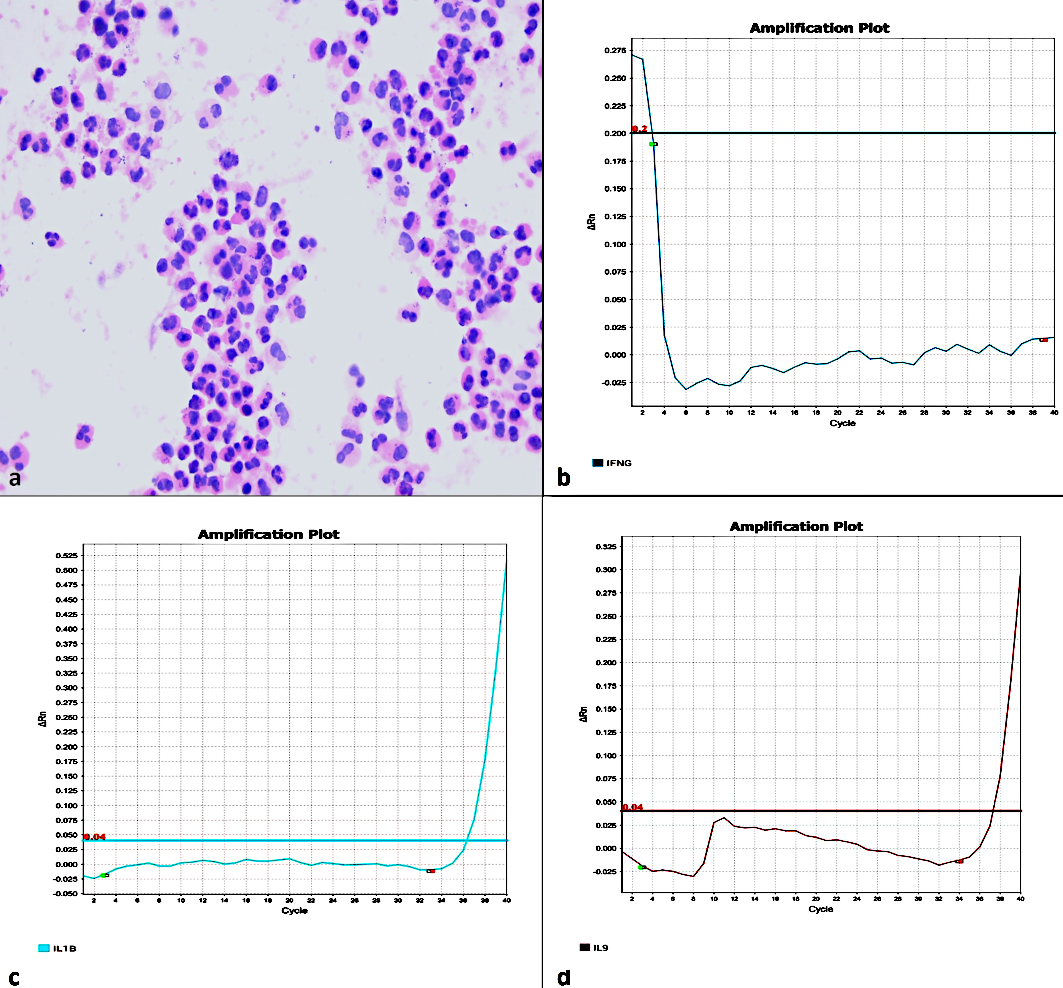

Supplement: Supplementary file 2 — Additional file 2. Explanatory case 1, showing a high number of neutrophils (a, hematoxylin and eosin staining, 40x original magnification) in BAL of a CARD patient without superinfection. IFN-γ was not detected while IL1β and IL-9 were found by molecular analyses (b, c and d, respectively). [file 12931_2023_2464_MOESM2_ESM.docx]

# Additional Figure 2


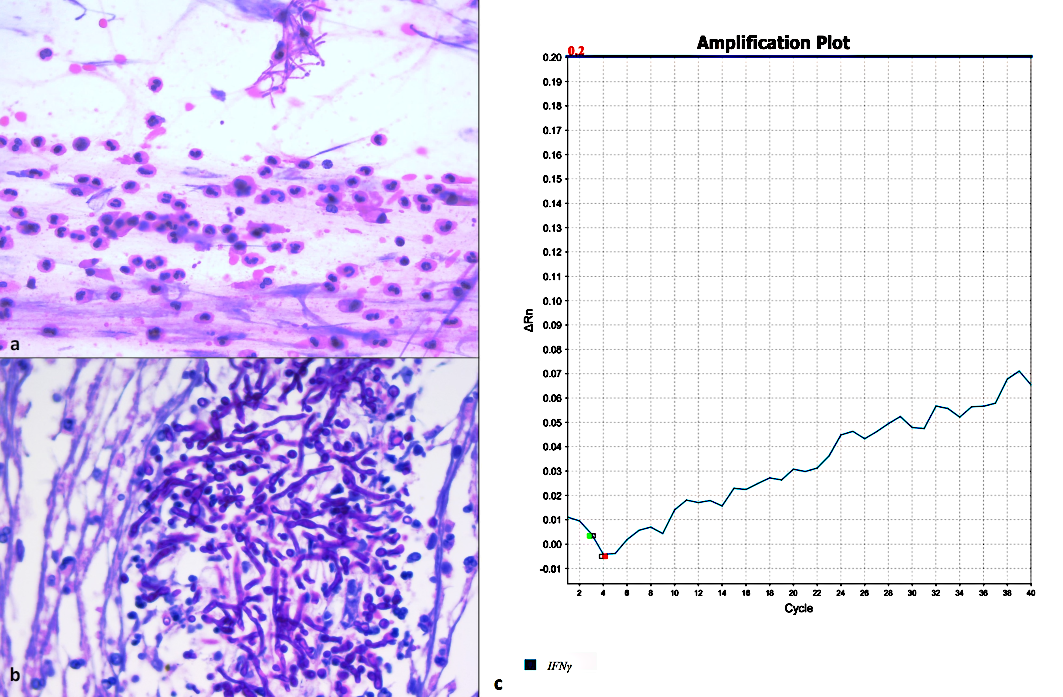

Supplement: Supplementary file 3 — Additional file 3. Explanatory case 2, showing a high number of neutrophils (a, hematoxylin and eosin staining, 40x original magnification) in BAL of a CARDS patient with a concurrent Aspergillus infection and numerous hyphae well seen at high magnification with special stain (b, PAS staining, 40x original magnification). IFN-γ was not detected by molecular analyses (c). [file 12931_2023_2464_MOESM3_ESM.docx]
